# Comparing an eHealth Program (My Hip Journey) With Standard Care for Total Hip Arthroplasty: Randomized Controlled Trial

**DOI:** 10.2196/22944

**Published:** 2021-03-03

**Authors:** Rosemary Saunders, Karla Seaman, Laura Emery, Max Bulsara, Catherine Ashford, Judith McDowall, Karen Gullick, Beverley Ewens, Trudy Sullivan, Charlotte Foskett, Lisa Whitehead

**Affiliations:** 1 School of Nursing and Midwifery Edith Cowan University Joondalup Australia; 2 Institute for Health Research The University of Notre Dame Fremantle Australia; 3 Hollywood Private Hospital Perth Australia; 4 Department of Preventive and Social Medicine University of Otago Dunedin New Zealand

**Keywords:** hip arthroplasty, education, eHealth program, rehabilitation, economic evaluation

## Abstract

**Background:**

The role of eHealth programs to support patients through surgical pathways, including total hip arthroplasty (THA), is rapidly growing and offers the potential to improve patient engagement, self-care, and outcomes.

**Objective:**

The aim of this study is to compare the effects of an eHealth program (intervention) versus standard care for pre- and postoperative education on patient outcomes for primary THA.

**Methods:**

A prospective parallel randomized controlled trial with two arms (standard care and standard care plus access to the eHealth education program) was conducted. Participants included those who underwent THA. Outcome measures were collected preadmission, at 6 weeks, and at 3 and 6 months after surgery. The primary outcome was the Hip Dysfunction and Osteoarthritis Outcome Score. Secondary outcomes were a 5-level 5-dimension quality of life measure and the self-efficacy for managing chronic disease scale. Demographic and clinical characteristics were also collected. A satisfaction survey was completed by all participants 6 weeks after surgery, and those in the intervention arm completed an additional survey specific to the eHealth program.

**Results:**

A total of 99 patients were recruited: 50 in the eHealth program (intervention) and 49 in standard care (control). Clinical improvements were demonstrated in both groups across all time points. Per-protocol analysis demonstrated no differences between the groups for all outcome measures across all time points. Participants in the eHealth program reported that the program was accessible, that they felt comfortable using it, and that the information was helpful.

**Conclusions:**

This study demonstrated that the eHealth program, in addition to standard care, had no additional benefit to THA recovery compared with standard care alone. The study found that the eHealth program was highly valued by participants, and it supported the preoperative preparation, recovery, and postoperative rehabilitation of participants.

**Trial Registration:**

Australian New Zealand Clinical Trial Registry ACTRN12617001433392; http://www.anzctr.org.au/Trial/Registration/TrialReview.aspx?id=373657

## Introduction

eHealth programs can provide individualized patient care at the preoperative, perioperative, and postoperative stages and have the potential to improve patient engagement, self-care, and outcomes across the surgical pathway [1,2]. The implementation of eHealth has many benefits, including enabling a single source of information that can be regularly and easily updated within a rapidly changing environment and enabling equitable access to all patients regardless of geographical location. Various capabilities can be incorporated into eHealth programs, including platforms to communicate directly with health professionals and electronic reminders to prompt patients to complete an exercise or take medication, and it can also be used by other health professionals and caregivers to provide an enhanced continuity of care [2].

One surgical pathway where preadmission, perioperative, and postoperative education is essential is total hip arthroplasty (THA) to prepare people physically and psychologically before surgery and to promote recovery after surgery. THA is a surgical procedure that improves both joint function and quality of life (QoL) in patients with hip osteoarthritis [3]. Osteoarthritis is a major disabling joint disorder worldwide, with the hip being the second most affected joint, and can result in pain, decreased function, and reduced QoL [4]. Within Australia and internationally, the number of people undergoing THA has increased annually over the last 10 years [5,6]. In Australia, over half of all hip arthroplasties (59.7%) are conducted in private hospitals [3].

The most prevalent form of education delivery for THA currently includes a combination of one-to-one verbal discussions, patient group sessions, educational booklets, and educational videos [7]. Many studies and reviews have demonstrated the benefits of these education programs, including reduced length of hospital stay, lower readmission rates, fewer adverse events, increased functional abilities, improved QoL, less anxiety, more effective pain management, and improved cost-effectiveness [8-12].

The incorporation of eHealth programs in the delivery of education has shown some potential to further enhance the educational experience and outcomes for postsurgical rehabilitation for orthopedic patients, including those undergoing THA [13]. Most studies have focused on the use of telerehabilitation in either the pre- or postsurgical periods [1,13-15]. A systematic review conducted on the evidence of the benefit of telerehabilitation after orthopedic surgery has shown strong to moderate grades of evidence for hip replacement interventions; the review recommends that high-methodological quality studies are needed [13]. Therefore, this study adds to the body of knowledge by conducting a high-quality randomized controlled trial (RCT) that aims to investigate the use of telerehabilitation across the perioperative period and not only the rehabilitation phase and compares the addition of an eHealth program (intervention) versus standard care (control) for pre- and postoperative education on patient outcomes for primary THA.

## Methods

### Study Design

A prospective RCT was conducted in a private metropolitan hospital in Western Australia. The trial consisted of two arms: one receiving the eHealth program and standard care (intervention) and the other receiving only standard care (control).

### Participants

Participants included patients undergoing primary elective THA in a private hospital. Patients were included if they were (1) 18 years or older, (2) able to provide informed consent, and (3) had at least three weeks’ lead-up time before THA surgery. Exclusion criteria included (1) admission to undergo a THA revision, a bilateral THA, THA following a fractured neck of the femur, or a previous THA; (2) inability to write or speak in English; (3) no access to a web-based device; and (4) a risk assessment and prediction tool score less than 6.

### Recruitment

Participants were screened and invited to hear more about the study by the preadmission nurse during the routine preadmission phone call. Eligible participants were then provided with additional study information and invited to participate by a member of the research team. The recruitment for the study was conducted from January 2018 to January 2019.

### Randomization

Participants were randomized one-to-one using permuted block randomization to ensure that an equal number of participants were allocated to each arm of the trial. Allocation concealment in the order of recruitment was conducted *off site* after consent had been obtained by a researcher, separate to participant recruitment. Blinding of the participant or health care team was not possible due to the type of intervention.

### Standard Care

The standard practice was an enhanced recovery program (ERP) based on an orthopedic recovery program developed in the United Kingdom [16]. The ERP included an enhanced recovery booklet received before admission; a 1-hour, hospital-based, face-to-face preoperative education session presented by a registered nurse, occupational therapist, pharmacist, and physiotherapist; and follow-up phone call post discharge. The program included information and education to support patients to prepare for hospital, during hospital, discharge, and post discharge.

### Intervention

Participants in the intervention arm received standard care plus access to the *My Hip Journey* eHealth education program. Depending on the participant’s surgical approach (posterior, anterior, or SUPERPATH), which was determined by the surgeon’s discretion, they were allocated into 1 of 3 types of programs. Access to the program was provided at least 2 weeks before surgery, and the program was run until 6 weeks post surgery.

*My Hip Journey* provided participants with web-based access to a range of educational resources, including fact sheets, videos, exercise videos, and email reminders about the pre- and postoperative care of a THA. Participants were encouraged to log in daily to view their *My Program* window displaying a list of videos and information as well as exercises that had been allocated for them to view or complete that day. Participants could also communicate with the health care team at the hospital using the communication log within the program; they could also invite other health care professionals or support persons to be part of the program.

### Data Collection

Participants completed data collection electronically in four phases: (1) preadmission, (2) 6 weeks, (3) 3 months, and (4) 6 months after surgery. Across all the four phases, participants completed the primary outcome measure Hip Dysfunction and Osteoarthritis Outcome Score (HOOS) and the secondary outcome measures of EuroQoL 5-Dimension 5-Level (EQ-5D-5L) and self-efficacy for managing chronic disease (SEMCD). The EQ-5D-5L consists of 2 parts, the EQ-5D visual analogue scale (VAS) and the index score, which are scored 0-100 and 0-1. At 6 weeks postsurgery (phase 2), participants also completed a satisfaction survey, and those in the intervention arm completed an additional survey specific to the eHealth program, and web-based analytics were also sourced. Further information on the data collection tools is outlined in a protocol paper [17].

### Sample Size

Sample size calculations were conducted based on the primary outcome (HOOS). The calculations were conducted for 3 out of the 5 HOOS subscales, and the QoL subscale required the largest sample size with a minimal clinically important improvement of 17 [18] and a SD of 23.5 [19]. On the basis of a power of 90% and a 5% significance level, 42 participants per group were required. A sample size of 50 per group was required to allow for a dropout rate of approximately 15%. Therefore, the estimated and required sample size for this study was 100 participants.

### Statistical Analysis

Data were reported in accordance with the CONSORT (Consolidated Standards of Reporting Trials). The mean (SD) and percentages were used to describe the characteristics of the study group and survey responses. The categorical responses for the 5 dimensions of the EQ-5D-5L (mobility, self-care, usual activities, pain/discomfort, and anxiety/depression) for each participant were transformed into an index score using the UK EQ-5D-5L value set [20].

Independent sample *t* test and chi-square or Fisher exact tests were conducted to determine any differences in baseline characteristics. Independent *t* tests were used to examine the differences in baseline outcome scores. Treatment effects were calculated on the pre- to postintervention outcomes at 6 weeks using an independent sample *t* test to examine the differences between groups. Further analysis was performed on the posttreatment effects at 3 and 6 months postsurgery. The clinical treatment effect of each intervention group was further analyzed using multilevel mixed-effects linear regression pre- to postintervention changes across the range of outcome measures to account for repeated measures with the covariates of age and gender.

## Results

In total, 99 participants were recruited for the study, with 50 allocated to the intervention group and 49 to the control group. Two participants withdrew because their surgery was postponed when their private health fund did not cover THA, leaving 47 participants in the control group.

**Figure 1:**
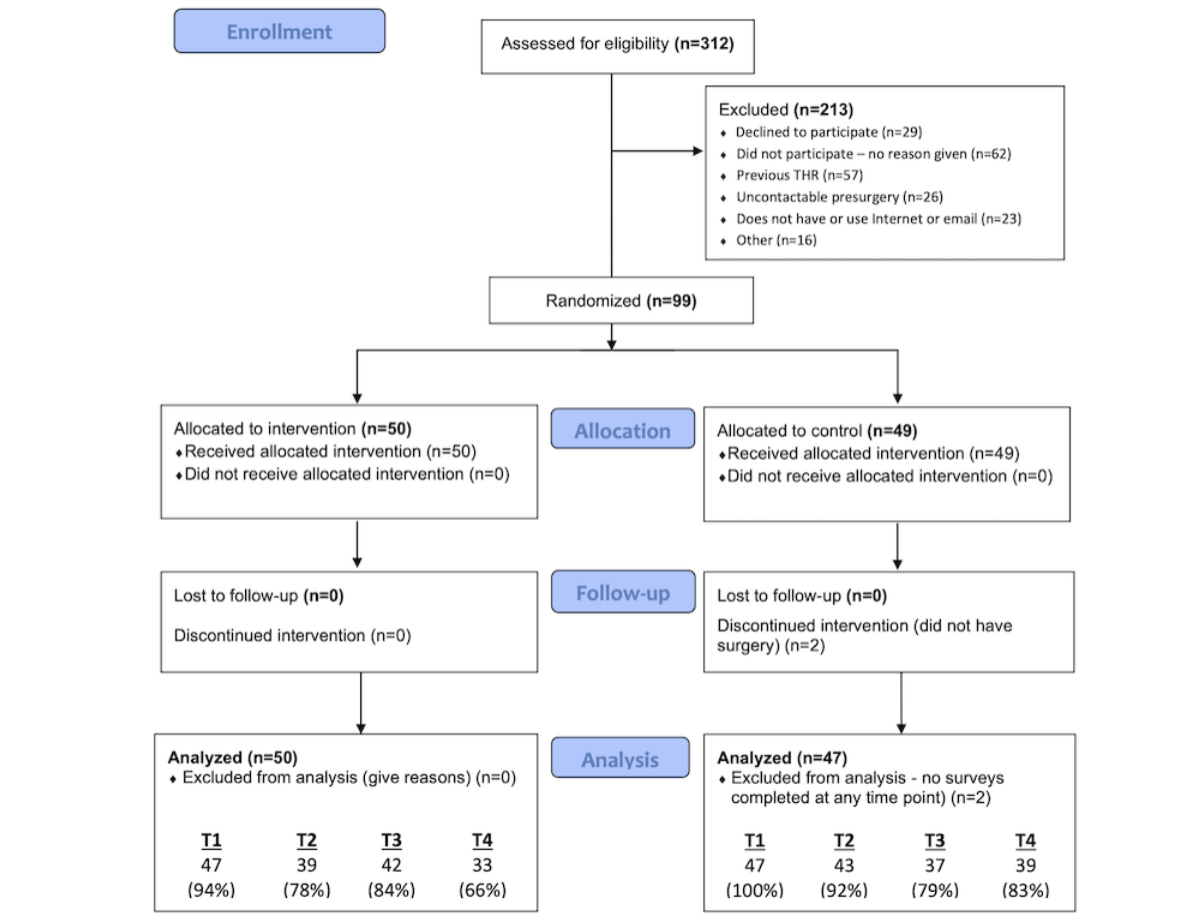
CONSORT (Consolidated Standards of Reporting Trials) flow diagram of patients participating in the study.

Data collection commenced in January 2018 and was completed in July 2019. Loss to follow-up occurred during each phase of the study. At the end of phase 4 (6-month follow-up), 66% (33/50) of participants remained in the intervention group and 82.9% (39/49) in the control group. A flow diagram of the patients participating in this study is outlined in the CONSORT diagram (Figure 1).

### Baseline Demographics and Clinical Characteristics

No significant difference was found between the control and intervention groups in terms of baseline demographic data and clinical characteristics (type of surgery and length of hospital stay; Table 1). A statistically significant difference at baseline for pain and activities of daily living was found between the groups for the HOOS scores (Table 2).

**Table 1:** Baseline demographics of participants in the intervention and control groups.

Characteristics		Intervention (n=50)	Control (n=49)	*P* value
**Gender, n (%)**				.76^a^
	Male	26 (52)	24 (49)	
	Female	24 (48)	25 (51)	
Age (years), mean (SD)		61.7 (12.1)	64.6 (9.7)	.20^b^
**Age (years), n (%)**				.69^a^
	60	20 (40)	16 (33)	
	61-70	17 (34)	17 (34)	
	≥71	13 (26)	16 (33)	
RAPT^c^ score, mean (SD)		10.5 (1.2)	10.6 (1.3)	.79^b^
**RAPT score, n (%)**				>.99^d^
	Additional interventions needed before discharge directly home (score 6-9)	7 (14)	6 (12)	
	Discharge directly home (score 10-12)	43 (86)	42 (86)	
	Missing	0	1 (2)	
**Type of hip surgery, n (%)**				.42^d^
	Left THR^e^	21 (42)	22 (45)	
	Right THR	29 (58)	25 (51)	
	No surgery	0	2 (4)	
**Surgery approach, n (%)**				.18^d^
	Posterior	26 (52)	19 (39)	
	Anterior	23 (46)	24 (49)	
	SUPERPATH	1 (2)	4 (8)	
	No surgery	0	2 (4)	
Length of stay (days), mean (SD)		3.9 (1.5)	3.7 (1.2)	.46^b^
Attended education class, n (%)		25 (50)	26 (53)	.76^a^
Known readmission, n (%)		4 (8)	2 (4)	.68^d^

^a^Chi-square test.

^b^Independent *t* test.

^c^RAPT: risk assessment and prediction tool.

^d^Fisher exact test.

^e^THR: total hip replacement.

**Table 2:** Primary outcome assessment Hip Dysfunction and Osteoarthritis Outcome Score.

Time periods		Intervention		Control		Difference	
		Mean (SD)	n (%)	Mean (SD)	n (%)	*d* (95% CI)	*P* value
**Baseline/preadmission^a^**							
	Symptoms	49.48 (18.28)	47 (100)	43.38 (20.34)	47 (100)	6.10 (−1.82 to 14.02)	.13
	Pain	51.49 (12.41)	47 (100)	45.33 (14.07)	46 (98)	6.16 (0.70 to 11.62)	*.03* ^b^
	Activities of daily living	56.44 (15.50)	47 (100)	47.78 (14.86)	47 (100)	8.67 (2.44 to 14.88)	*.007*
	Sport/recreation	35.99 (23.45)	47 (100)	30.34 (19.93)	46 (98)	5.65 (−3.32 to 14.62)	.21
	Quality of life	30.72 (17.57)	47 (100)	27.26 (16.09)	47 (100)	3.46 (−3.44 to 10.36)	.32
**Change baseline—6 weeks^a^**							
	Symptoms	27.42 (21.01)	37 (95)	31.62 (25.17)	41 (95)	−4.20 (−14.71 to 6.32)	.43
	Pain	30.07 (19.41)	37 (95)	33.00 (18.67)	40 (95)	−2.93 (−11.58 to 5.72)	.50
	Activities of daily living	23.43 (16.23)	37 (95)	27.44 (20.38)	41 (95)	−4.02 (−12.39 to 4.35)	.34
	Sport/recreation	18.24 (26.49)	37 (95)	21.19 (30.9)	40 (95)	−2.95 (−16.07 to 10.16)	.66
	Quality of life	28.72 (21.72)	37 (95)	27.74 (26.45)	41 (95)	0.97 (−10.01 to 11.96)	.86
**Change baseline—3 months^a^**							
	Symptoms	32.68 (23.01)	39 (93)	37.53 (28.41)	37 (100)	−4.85 (−16.64 to 6.94)	.42
	Pain	36.6 (17.5)	39 (93)	39.65 (21.62)	36 (97)	−3.05 (−12.07 to 5.97)	.50
	Activities of daily living	28.56 (18.67)	39 (93)	35.13 (23.35)	37 (100)	−6.57 (−16.21 to 3.07)	.18
	Sport/recreation	32.53 (30.29)	39 (93)	39.70 (32.01)	36 (97)	−7.17 (−21.50 to 7.17)	.32
	Quality of life	39.42 (24.72)	39 (93)	42.91 (26.4)	37 (100)	−3.48 (−15.16 to 8.19)	.55
**Change baseline—6 months^a^**							
	Symptoms	34.36 (22.97)	32 (97)	41.25 (24.61)	39	−6.89 (−18.25 to 4.48)	.23
	Pain	38.52 (19.01)	32 (97)	40.72 (18.88)	38	−2.21 (−11.27 to 6.86)	.63
	Activities of daily living	30.71 (19.50)	32 (97)	37.55 (19.76)	39 (100)	−6.84 (−16.19 to 2.50)	.15
	Sport/recreation	40.04 (31.74)	32 (97)	44.19 (28.30)	38	−4.15 (−18.47 to 10.17)	.57
	Quality of life	45.12 (27.39)	32 (97)	47.44 (22.75)	39 (100)	−2.34 (−14.19 to 9.55)	.70

^a^Independent *t* test.

^b^*P* value in italics are statistically significant.

### HOOS

From the analysis of the HOOS scores, it was found that both groups improved immediately after surgery, and this improvement was demonstrated across all 5 HOOS domains. Participants continued to improve at 3 months and 6 months after surgery. Baseline scores for pain and activities of daily living were significantly different between the intervention and control groups. No significant differences in changes between the intervention and control groups were detected at baseline and at 6 weeks, at 3 months, and at 6 months after surgery for the HOOS scores (Table 2).

### EuroQol EQ-5D-5L

An improvement in health-related quality of life (HRQoL) was observed at 6 weeks, 3 months, and 6 months after surgery in both the control and intervention groups, as measured by the EQ-5D-5L VAS and the EQ-5D-5L index scores (Table 3). However, there were no statistically significant differences between the groups at any of the time points.

**Table 3:** Secondary outcome assessment 5-level 5-dimension quality of life measure.

Time periods		Intervention		Control		Difference	
		Mean (SD)	n (%)	Mean (SD)	n (%)	*d* (95% CI)	*P* value
**Baseline/preadmission^a^**							
	Index	0.64 (0.19)	47 (100)	0.59 (0.21)	47 (100)	0.05 (−0.03 to 0.14)	.20
	VAS^b^	64.79 (20.02)	47 (100)	68.55 (18.23)	47 (100)	−3.77 (−11.62 to 4.09)	.34
**Change baseline—6 weeks^a^**							
	Index	0.17 (0.20)	37 (95)	0.19 (0.26)	42 (98)	−0.02 (−0.12 to 0.09)	.77
	VAS	10.43 (15.05)	37 (95)	10.52 (15.73)	42 (98)	−0.09 (−7.01 to 6.83)	.98
**Change baseline—3 months^a^**							
	Index	0.21 (0.21)	40 (95)	0.23 (0.21)	37 (100)	−0.02 (−0.11 to 0.08)	.72
	VAS	12.82 (20.08)	39 (93)	9.76 (15.05)	37 (100)	3.06 (−5.08 to 11.21)	.46
**Change baseline—6 months^a^**							
	Index	0.22 (0.18)	32 (97)	0.27 (0.22)	39 (100)	−0.05 (−0.15 to 0.05)	.29
	VAS	15.77 (16.90^c^)	31 (94)	13.23 (13.21)	39 (100)	2.54 (−4.63 to 9.72)	.48

^a^Independent *t* test.

^b^VAS: visual analogue scale.

^c^Large outlier (−62)—intervention participant (ID#51) not included.

### SEMCD

Both groups reported an increased sense of postsurgical self-efficacy. Both groups had an above-average level of self-efficacy preoperatively. Participants continued to improve at 3 months and 6 months after surgery. However, no significant differences in changes between the intervention and control groups were detected at baseline and at 6 weeks, at 3 months, and at 6 months after surgery (Table 4).

**Table 4:** Secondary outcome assessment self-efficacy for managing chronic disease score; per-protocol analysis.

Time Periods	Intervention		Control		Difference between groups	
	Mean (SD)	n (%)	Mean (SD)	n (%)	*d* (95% CI)	*P* value
Baseline/preadmission^a^	6.52 (1.88)	47 (100)	6.60 (1.80)	47 (100)	−0.07 (−0.82 to 0.69)	.86
Change baseline—6 weeks^a^	1.29 (2.09)	37 (35)	1.51 (1.70)	42 (98)	−0.22 (−1.06 to 0.63)	.62
Change baseline—3 months^a^	1.85 (2.06)	41 (98)	1.49 (1.67)	37 (100)	0.36 (−0.49 to 1.21)	.40
Change baseline—6 months^a^	2.06 (2.24)	32 (97)	1.76 (1.61)	39 (100)	0.31 (−0.61 to 1.22)	.51

^a^Independent *t* test.

### Repeated Measures Analysis

A repeated measure analysis based on per-protocol analysis was performed using multilevel mixed-effects linear regression accounting for age and gender. We found no effect over time with the interaction of intervention by time, considering any differences in baseline measures. Thus, the results were the same regardless of the intervention group.

### Economic Evaluation

As there were no statistically significant differences in the primary and secondary outcomes for the eHealth program and standard care, no further economic analysis was conducted.

### Satisfaction Survey Results

The satisfaction survey was administered electronically 6 weeks after surgery to all participants, with 43 participants in the control group and 39 participants in the intervention group completing the survey and 92% and 78% response rate, respectively. Across all 6 questions, no statistically significant difference in the satisfaction levels between the intervention and control groups was noted (Table 5). The majority of participants either strongly agreed or somewhat agreed that the information was easy to follow (intervention group: 39/39, 100%; control group: 40/43, 93%), found the presurgery information helpful (intervention group: 39/39, 100%; control group: 40/43, 93%), found the postsurgery information helpful (intervention group: 37/39, 94.8%; control group: 39/43, 90.7%), and the content gave me a good understanding of my surgery pathway (intervention group: 38/39, 97.4%; control group: 37/43, 86%), The majority of participants either strongly agreed or somewhat agreed that the content gave me a good understanding of how to maximize recovery (intervention group: 37/39, 94.8%; control group: 38/43, 88.4%), and I feel that the package that was supplied assisted me in my recovery (intervention group: 36/39, 92.3%; control group: 37/43, 86%).

**Table 5:** Satisfaction survey results by group.

Survey questions		Control group (n=43), n (%)	Intervention group (n=39), n (%)	*P* value^a^
**I found the information easy to follow**				.34
	Strongly disagree	0 (0)	0 (0)	
	Somewhat disagree	2 (4.6)	0 (0)	
	Neither agree nor disagree	1 (2.3)	0 (0)	
	Somewhat agree	11 (25.6)	8 (20.5)	
	Strongly agree	29 (67.4)	31 (79.5)	
**I found the presurgery information helpful**				.15
	Strongly disagree	3 (7.0)	0 (0)	
	Somewhat disagree	0 (0)	0 (0)	
	Neither agree nor disagree	0 (0)	0 (0)	
	Somewhat agree	12 (27.9)	8 (20.5)	
	Strongly agree	28 (65.1)	31 (79.5)	
**I found the postsurgery information helpful**				.51
	Strongly disagree	2 (4.65)	0 (0)	
	Somewhat disagree	0 (0)	1 (2.6)	
	Neither agree nor disagree	2 (4.65)	1 (2.6)	
	Somewhat agree	12 (27.9)	10 (25.6)	
	Strongly agree	27 (62.8)	27 (69.2)	
**The content gave me a good understanding of my surgery pathway**				.42
	Strongly disagree	1 (2.3)	0 (0)	
	Somewhat disagree	2 (4.6)	0 (0)	
	Neither agree nor disagree	3 (7)	1 (2.6)	
	Somewhat agree	5 (11.6)	6 (15.4)	
	Strongly agree	32 (74.4)	32 (82)	
**The content gave me a good understanding on how to maximize recovery**				.70
	Strongly disagree	2 (4.6)	0 (0)	
	Somewhat disagree	1 (2.3)	1 (2.6)	
	Neither agree nor disagree	2 (4.6)	1 (2.6)	
	Somewhat agree	11 (25.6)	10 (25.6)	
	Strongly agree	27 (62.8)	27 (69.2)	
**I feel that the package that was supplied assisted me in my recovery**				.80
	Strongly disagree	2 (4.7)	1 (2.6)	
	Somewhat disagree	0 (0)	0 (0)	
	Neither agree nor disagree	4 (9.3)	2 (5.1)	
	Somewhat agree	12 (27.9)	10 (25.6)	
	Strongly agree	25 (58.1)	26 (66.7)	

^a^Chi-square test.

Over 80% (51/82) of the participants in both the intervention and control groups responded to the open-ended questions. Most of the intervention group (n=16) stated that there was no further information that they needed, and they felt *well informed*. In contrast, others (n=11) reported a lack of information pertaining to the expected physical abilities after the surgery and weaning off crutches and suggested including additional videos from physiotherapists and occupational therapists along with more information on anesthetic options, medications, and risks associated with surgery. One participant suggested that the program should have additional information for people living without a support person. In addition, many participants in the control group (n=15) provided suggestions for additional information, including the need for additional occupational therapy and physiotherapy advice, information on presurgery exercise, postsurgery exercises, and the recovery pathway. Other feedback was specific to the individual participant experiences and included suggestions for further information about medication, postoperative complications, and variations in length of stay.

### eHealth Program Survey

Participants in the intervention group completed an additional survey specific to the use of the eHealth program 6 weeks after surgery. In total, 39 participants completed the survey, of which 97% (n=38) accessed the program at least once. Participants accessing the program varied: 30% (11/37) used it daily, 27% (10/37) used it 2-3 times a week, 13% (5/37) used it at least once a fortnight, and 30% (11/37) only accessed the program a couple of times overall (less than once a fortnight). The majority felt that the “application was easy to use” (35/37, 95%,), they felt “comfortable using the application” (35/37, 95%), it was “easy to find the information needed” (35/37, 95%), “the organization of the information on the application screen was clear” (35/37, 95%), the “information was effective in helping them complete the daily tasks” (33/37, 89%), and the “content in the emails were helpful” (33/37, 89%). All participants said they somewhat agreed or agreed that they would recommend the app to others; however, some participants stated that they would still prefer paper-based information. Most participants were satisfied with the app (33/37, 89%). Only a small percentage of respondents contacted the health professional using the email within the app (7/37, 18%), of which 3 neither agreed nor disagreed that the health professional’s response supported them in their recovery, 1 somewhat agreed, and 3 agreed that the responses supported them in their recovery.

In response to what participants liked most about the program, the majority provided feedback (n=31), including ease of access to the information (n=10) and the information provided was informative, concise, and clearly presented (n=10). Others commented on the benefits of the program through videos, exercise videos, clear layout of the program, environmentally friendly program, reinforcing good day-to-day practice, and benefit of using in your own time. A total of 17 participants shared dislikes of the program related to repetition of information, frequency of emails, and timing of information. Moreover, 19 participants provided additional comments, with the majority (n=15) sharing positive feedback, including “it was an excellent tool to assist my recovery,” “the information provided kept me informed,” “wonderful resource” and “it made me very well informed for my surgery.” Other participants (n=4) provided further feedback, including the importance of including a social worker, having more practical advice from an occupational therapist, having too many boxes to record their daily activities, and their lack of confidence in technology affected their use of the program.

### Safety and Adverse Events

For all participants involved in the study, there were a total of 6 readmissions to hospitals, 4 from the eHealth program, and 2 from standard care. Reasons for readmission included revision of the hip, excision trochanter bursa, gluteal tendon repair, washout of THA, dislocation of THA with revision, and development of deep vein thrombosis in the leg. Two participants from the intervention group transitioned to the rehabilitation ward following surgery for further in-hospital support.

## Discussion

### Principal Findings

ERPs for patients undergoing THA have become increasingly common and have been shown to reduce hospital length of stay and complications [21]. Preoperative patient education is a key part of ERP protocols, and health care facilities are exploring eHealth as a flexible option to support patient education and enhance patient involvement. This study used an eHealth program for pre- and postoperative education for THA and found it to be as effective as standard care. Participants in both groups demonstrated improvement in the primary outcome measure (HOOS) at 6 weeks, 3 months, and 6 months after surgery. No statistically significant differences were observed between the intervention and control groups. Other studies have also compared the effectiveness and benefits of eHealth apps in joint arthroplasty and reported similar findings [22-24]. Across the secondary outcomes of length of stay, HRQoL, and SEMCD, no statistically significant difference between the intervention and control groups was observed. Self-reported HRQoL increased in both groups after surgery, which was consistent with other studies reporting improved QoL with increased functionality [25].

Preoperative patient education was identified as being important in contributing to patient recovery by providing patients with more realistic expectations and an understanding of the postoperative period while empowering them to be actively engaged in their recovery [5,26]. Patient satisfaction with both the standard care education and the eHealth program was high, and there was no significant difference between the groups. More specifically, the presurgery education information and postsurgery information were found to provide a good understanding of the surgical journey and of how to maximize recovery. Previous studies also found positive benefits of preoperative patient education in THA [6,27]. Participants in the intervention group reported high satisfaction scores for the eHealth program, in the helpfulness of the pre- and postsurgery information, and for the content supporting their understanding of the surgery and maximizing their recovery. In addition, most participants stated that there was no further information that they needed, and they felt *well informed*. This was an important finding as a key part of ERPs for hip arthroplasty was pre- and postoperative patient education, particularly exercises, to achieve functional recovery and reduced hospital stay [28]. Constructive feedback from both groups identified areas for development in patient education, with specific feedback from the control group on the need for more information on pre- and postsurgery exercises. This was not reported by the intervention group, and the exercise videos in the eHealth program most likely addressed this need, but they did suggest including additional information about preoperative preparation within the presurgery videos.

Health professionals have identified that a patient’s knowledge of postoperative exercises and undertaking these exercises correctly contributes to the success of hip arthroplasty, and eHealth apps can facilitate better patient engagement with the discharge exercise regime [29]. The overall satisfaction with using the eHealth program was positive and the regular use (at least once per week) by most of the participants may have contributed to the perceived benefits. Over 75% reported positive benefits focused on ease of use, including good visual display, access via any device, quality of information through the daily email reminders, web-based resources and videos encouraging regular use, and flexibility. These reported benefits align with the usefulness, utility, and usability (including learnability, memorability, and satisfaction) criteria identified for usable eHealth programs [30].

Participants’ individual differences and preferences formed the basis of suggestions for improvement, including considering the frequency of emails, the volume of information, and the need for a dedicated focus on recovery for people who have limited or no care support. Overall, the positive feedback identified that the program was a valuable resource in supporting patient recovery, and participants would recommend the use of the program to others. These findings support the notion that developing effective eHealth programs requires feedback from end users and recognizes the value in supporting patient engagement in their own recovery [31,32].

Interestingly, only a few participants reported using the functionality to contact other health professionals or hospitals via email. This may indicate that the platform provided sufficient information to support recovery, and urgent concerns may have been directed to the surgeon. This area could be explored further, and the program expanded to include discharge plans on the platform as a record for patients and to communicate directly with the primary health care team.

On the basis of the findings of this study, it is recommended that the eHealth program be provided as an option to support patients in their perioperative journey for hip arthroplasty. The results of this study can help inform the development and future research of telerehabilitation programs for other surgical procedures.

### Limitations

The study was developed and conducted according to the CONSORT statement. This study had two key limitations. The study was conducted in an acute private hospital; hence, the findings may not be generalizable to other hospitals because the results are limited to the study population and may not be representative of participants at other hospitals. The study may have lent itself to participants who were more comfortable with technology, which may have created a potential selection bias.

### Conclusions

Preoperative patient education is important for positive patient outcomes following hip arthroplasty, and eHealth patient education is becoming an increasingly flexible option to deliver these resources to patients and guide the preparation and recovery from surgery along with their direct contact with health care professionals. This study demonstrated that participants in the intervention group did not differ in outcome measures compared with the control group, who received standard patient education. The study demonstrated that an eHealth program created an opportunity to provide preoperative guidance on preparation and recovery and supported postoperative rehabilitation. The acceptance of the program was high, with participants reporting that it was easy to use and enabled them to access information when they wanted to. These promising results demonstrate that health care organizations can implement and adapt digital health systems with good uptake by patients. Larger studies would help further inform how eHealth programs can be adapted for other orthopedic and surgical procedures.

## Appendix

Multimedia Appendix 1 CONSORT-eHEALTH checklist (V 1.6.1).

## Abbreviations

ERP: enhanced recovery program
EuroQol EQ-5D-5L: 5-level 5-dimension quality of life measure
HOOS: Hip Dysfunction and Osteoarthritis Outcome Score
HRQoL: health-related quality of life
QoL: quality of life
RCT: randomized controlled trial
SEMCD: self-efficacy for managing chronic disease
THA: total hip arthroplasty
VAS: visual analogue scale
